# Downregulation of a Mitochondrial NAD^+^ Transporter (NDT2) Alters Seed Production and Germination in Arabidopsis

**DOI:** 10.1093/pcp/pcaa017

**Published:** 2020-02-17

**Authors:** Elias Feitosa-Araujo, Izabel de Souza Chaves, Alexandra Florian, Paula da Fonseca-Pereira, Jorge Alberto Condori Apfata, Elmien Heyneke, David Barbosa Medeiros, Marcel Viana Pires, Tabea Mettler-Altmann, H Ekkehard Neuhaus, Ferdinando Palmieri, Wagner L Ara�jo, Toshihiro Obata, Andreas P M Weber, Nicole Linka, Alisdair R Fernie, Adriano Nunes-Nesi

**Affiliations:** p1 Max Planck Partner Group, Departamento de Biologia Vegetal, Universidade Federal de Vi�osa, Vi�osa 36570-900, Minas Gerais, Brazil; p2 Max-Planck-Institute of Molecular Plant Physiology, Am M�hlenberg 1, Potsdam-Golm 14476, Germany; p3 Department of Plant Biochemistry, Heinrich Heine University D�sseldorf, D�sseldorf 40225, Germany; p4 Department of Plant Physiology, University of Kaiserslautern, Kaiserslautern 67663, Germany; p5 Department of Biosciences, Biotechnology and Biopharmaceutics, University of Bari, Bari 70125, Italy

**Keywords:** Metabolite transport, Mitochondria, Nicotinamide adenine dinucleotide, Seed germination, Seed production

## Abstract

Despite the fundamental importance of nicotinamide adenine dinucleotide (NAD^+^) for metabolism, the physiological roles of NAD^+^ carriers in plants remain unclear. We previously characterized the *Arabidopsis thaliana* gene (*At*1g25380), named *AtNDT2*, encoding a protein located in the mitochondrial inner membrane, which imports NAD^+^ from the cytosol using ADP and AMP as counter-exchange substrates for NAD^+^. Here, we further investigated the physiological roles of NDT2, by isolating a T-DNA insertion line, generating an antisense line and characterizing these genotypes in detail. Reduced *NDT2* expression affected reproductive phase by reducing total seed yield. In addition, reduced seed germination and retardation in seedling establishment were observed in the mutant lines. Moreover, remarkable changes in primary metabolism were observed in dry and germinated seeds and an increase in fatty acid levels was verified during seedling establishment. Furthermore, flowers and seedlings of *NDT2* mutants displayed upregulation of *de novo* and salvage pathway genes encoding NAD^+^ biosynthesis enzymes, demonstrating the transcriptional control mediated by NDT2 activity over these genes. Taken together, our results suggest that *NDT2* expression is fundamental for maintaining NAD^+^ balance amongst organelles that modulate metabolism, physiology and developmental processes of heterotrophic tissues.

## Introduction

Nicotinamide adenine dinucleotide (NAD^+^) is a central metabolite in cell metabolism, being involved in energy transactions and cellular signaling as well as acting as a coenzyme in various reactions (Rasmusson and Wallstr�m 2010, Gaki�re et�al. 2018). Unbalanced NAD^+^ metabolism not only results in changes in its levels but also in the general redox state of the cell, thereby impacting plant growth and development (Noctor et�al. 2006, Hashida et�al. 2009). Due to its ability to transport electrons, NAD^+^ is regarded as an essential compound in numerous redox reactions across the tree of life (Hashida et�al. 2010). However, changes in cellular redox state are particularly intrinsic to plant metabolism and are determined by the oxidation and reduction of various redox-active substances, especially NAD(H) and NADP(H) (Geigenberger and Fernie 2014). These pyridine nucleotides act as cofactors of many enzymes and as such represent some of the most interconnected metabolites within the cellular network. NAD(P)^+^ and its reduced form(s) NAD(P)H are involved in a wide range of key metabolic reactions including several in glycolysis, tricarboxylic acid (TCA) cycle, glycine decarboxylation, the Calvin–Benson cycle and the oxidative pentose phosphate pathway as well as in β-oxidation in peroxisomes (Bernhardt et�al. 2012, Geigenberger and Fernie 2014). Given that NAD^+^ and its derivatives participate in redox reactions, they play an important role in controlling the *in vivo* activities of dehydrogenase-catalyzed reactions. Despite the essentiality of NAD^+^ in all cellular organelles, the final steps of its biosynthesis are exclusively cytosolic in Arabidopsis (Noctor et�al. 2006, Hashida et�al. 2009). Thus, to perform its functions inside the mitochondrion, plastid, endoplasmic reticulum, Golgi complex and peroxisome, it must be imported into the organelles in a carrier protein-mediated process (Palmieri et�al. 2009).

Mitochondria are omnipresent in eukaryotic cells and play an important role in a myriad of physiological functions (Sweetlove et�al. 2010). In plants, mitochondria are involved in essential processes, such as (photo)respiration and energy supply to the cell, nitrogen assimilation, carbon metabolism and amino acid biosynthesis, as well as in response to stresses and intracellular signaling (Oswald et�al. 2001, Ara�jo et�al. 2012, Nunes-Nesi et�al. 2013). Unlike the external membrane, the inner membrane of mitochondria is impermeable to most metabolites. Thus, the existence of specific proteins is necessary to carry out the import and export of metabolites through this barrier (Millar and Heazlewood 2003, Haferkamp 2007, Palmieri et�al. 2011). The passage of hydrophobic compounds across the inner mitochondrial membrane is performed by a diverse range of proteins; predominant amongst them are the mitochondrial carrier family (MCF) proteins (Picault et�al. 2004). Members of this family are characterized as having a relatively small size, a molecular mass ranging from 30 to 34 kDa and the presence of six α-helix type transmembrane segments and five hydrophilic portions, which may be sub-divided into three domains (Picault et�al. 2004).

Mitochondrial transporters have largely been identified via biochemical studies in isolated mitochondria. These carriers were subsequently differentiated from one another based on their substrate specificities and relative sensitivities to various inhibitors (Palmieri et�al. 2011). Whilst some carriers are directly involved in oxidative phosphorylation, others are responsible for the transport of TCA cycle intermediates, amino acids or cofactors (Laloi 1999, Palmieri et�al. 2011, Palmieri 2013, Palmieri and Monn� 2016). One such MCF protein, NDT2, was identified and characterized as a mitochondrial NAD^+^ transporter, importing preferably NAD^+^ in strict counter exchange for ADP and AMP (Palmieri et�al. 2009). Two further NAD^+^ transporters have been described, a mitochondrial transporter, NDT1 (Chaves et�al. 2019), and a peroxisomal transporter, PXN (Agrimi et�al. 2012, Bernhardt et�al. 2012). The mitochondrial *AtNDT2* is strongly expressed in developing tissues and in cells with high metabolic activity, and its relative expression in comparison to *AtNDT1* varies according to the tissue concerned (Palmieri et�al. 2009, Chaves et�al. 2019). In mitochondria, despite NAD^+^ being the preferred substrate for NDT2, both NDT1 and NDT2 are capable of transporting, amongst others, adenylates, nicotinamide mononucleotide and nicotinic acid mononucleotide (Palmieri et�al. 2009). Promoter analysis indicated that *NDT2* is highly expressed in shoot apical meristem, veins of young and senescent leaves, developing siliques (including formation of the funicular structure), flower veins, developing pollen and the central cylinder of roots (Palmieri et�al. 2009). A recent study has, furthermore, provided the *in vivo* confirmation of our earlier *in vitro* observations that NDT2 transports NAD^+^, with mutants of this transporter containing lower mitochondrial NAD^+^ levels compared to the wild type (WT) (Luo et�al. 2019).

Despite a previous detailed molecular characterization of the NDT2 carrier (Palmieri et�al. 2009), the physiological function of this transporter remains poorly understood. For this reason, we isolated a T-DNA insertion mutant anda transgenic line with reduced expression of *NDT2* in Arabidopsis and performed a detailed physiological and biochemical characterization of both non-flowering plants and reproductive tissues of these genotypes. Our study reports the physiological characterization of a plant protein responsible for a mitochondrial NAD^+^ transport at the whole plant level and proves the importance of this transport for the normal operation of diverse cellular processes. The results are discussed both in the context of the importance of this transporter itself and intracellular co-ordination of plant metabolism in general. The data obtained are consistent with previous studies on pathways involved in NAD^+^ biosynthesis, highlighting the importance of this cofactor in reproductive function. However, in contrast to previous studies, we provide compelling evidence for the key role of this mitochondrial NAD^+^ transporter and for the implication of NAD^+^ compartmentation in the fine-tuning of normal reproductive and post-reproductive processes, including germination and seedling establishment.

## Results

A T-DNA insertion line with reduced expression of the gene encoding NAD^+^ mitochondrial transporter *At*NDT2 (*At*1g25380) was isolated. The T-DNA insertion position and primer binding sites used for the molecular characterization of the *NDT2* mutant are depicted in Fig.�1A. The T-DNA mutant line (GABI-Kat, GK_143G09) harbors a T-DNA insertion in the fifth exon. In addition to this mutant line, hereafter named *ndt2*^*−*^*:ndt2*^*−*^, we also used an antisense approach to obtain one line with reduced expression of *NDT2* (Fig.�1A). For this purpose, we generated the transgenic line by expressing the antisense (complementary) strand of DNA (cDNA) under the control of the *35S* promoter. After transformation, a total of 20 transgenic lines were selected based on their antibiotic resistance and their *NDT2* gene expression levels. Selected lines were used for seed production and further selection of lines was based solely on *NDT2* expression. Due to low *NDT2* expression in autotrophic tissues (Palmieri et�al. 2009), flowers were harvested for RNA extraction and analysis of *NDT2* expression. Following this analysis, we selected only the line with significant reduction in *NDT2* expression for further studies (Fig.�1B). The line *ndt2*^*−*^*:ndt2*^*−*^ displayed 45% of *NDT2* expression level found in WT, whilst the antisense line named *as-1-ndt2* presented 46% (Fig.�1B). Notably, plants with reduced *NDT2* expression showed no apparent phenotypes during their vegetative growth phase (Fig.�1C).

**Fig. 1:**
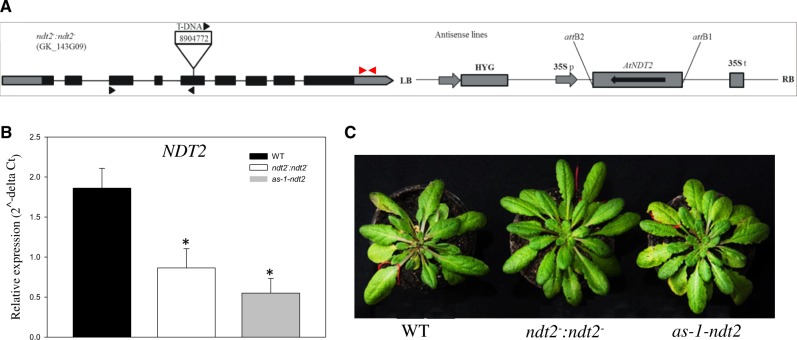
Isolation and characterization of genotypes deficient in the expression of the NAD^+^ mitochondrial transporter (*NDT2*) in *Arabidopsis thaliana* mutant lines. (A) Schematic representation of the gene *At*NDT2 (*At*1g25380) showing the T-DNA insertion site. The T-DNA insert, ∼4.5 kb, is not at scale. Boxes represent gene exons, black arrows on the T-DNA denote primer positions used for population screening and red arrows denote primers used for gene expression. The antisense construct includes the HYG, the 35S promoter, the gene *NDT2* in antisense position and the 35S terminator. (B) Expression by the qRT-PCR analysis of *NDT2* in flowers of the *A*. *thaliana* WT and mutant lines. Values are presented as mean � SE of determinations on six individual plants per line; asterisks indicate values that were determined by Student’s *t*-test to be significantly different (*P* < 0.05) from WT. (C) Phenotypic characterization of *A*. *thaliana* genotypes with the reduced expression of *NDT2*. The plants were 4 weeks old and grown in short-day conditions. HYG, hygromycin resistance gene.

### Effects of reduced expression of *AtNDT2* on the expression of genes involved in NAD^+^ metabolism in flowers and during the reproductive phase

Given that the *NDT2* gene is highly expressed in flowers, we aimed to evaluate the effects caused by the reduced expression of *NDT2* in the reproductive phase of *NDT2* mutants and WT plants. We first assessed the expression of the three NAD^+^ carrier genes, *AtNDT1*, *AtNDT2* and *AtPXN* in opened flowers of WT and *ndt2* plants. The relative transcript levels of *AtNDT1* increased in the *ndt2*^*−*^*:ndt2*^*−*^ and *as-1-ndt2* lines, whilst a slight increment in *AtPXN* expression was observed in the *as-1-ndt2* line (Fig.�2A). We next evaluated the expression of genes related to the de novo and salvage pathways of NAD^+^ biosynthesis in opened flowers to determine a possible influence of the reduced expression of *NDT2* on NAD^+^ metabolism genes during the reproductive phase. The de novo pathway in plants constitutes the enzymes aspartate oxidase (AO), quinolinate synthase (QS), quinolinate phosphoribosyltransferase (QPRT), nicotinate mononucleotide adenylyltransferase (NaMNAT) and NAD synthetase (NADS). Interestingly, the transcript levels of genes encoding these enzymes were higher in the *ndt2*^*−*^*:ndt2*^*−*^ and *as-1-ndt2* lines (Fig.�2B). Similarly, we determined the expression of genes encoding enzymes of the NAD^+^ biosynthetic salvage pathway as well as those related to NAD^+^ degradation. Genes encoding nicotinamidase 1 (NIC1), nicotinamidase 3, nicotinate phosphoribosyltransferase 1 and nicotinate phosphoribosyltransferase 2 exhibited elevated transcript levels in *ndt2* plants. The transcript levels of genes encoding NAD^+^ degrading enzymes poly(ADP-ribose)polymerases (PARP1 and PARP2) and nudix hydrolase (NUDIX7) were additionally evaluated. PARP1 levels were invariant across genotypes, whereas PARP2 transcript levels were significantly higher in the *as-1-ndt2* line (Fig.�2B). The increased expression of *NDT1* and *PXN* genes along with genes encoding enzymes of the NAD^+^ biosynthetic pathway might be explained as a compensation for the possible reduced NAD^+^ import into mitochondria in *ndt2* plants. We next evaluated the effects of reduced *NDT2* expression on reproductive parameters in *ndt2* and WT plants. Plants with reduced *NDT2* expression displayed a lower silique number and fewer seeds per silique, leading both conditions to a strong reduction in total seed production per plant (Fig.�2C). Furthermore, the silique length, silique width and the total seed weight were negatively affected in *ndt2* plants (Fig.�2C). Taken together, these phenotypic results demonstrate the pivotal role of NDT2 proteins to regulate the reproductive phase.

**Fig. 2:**
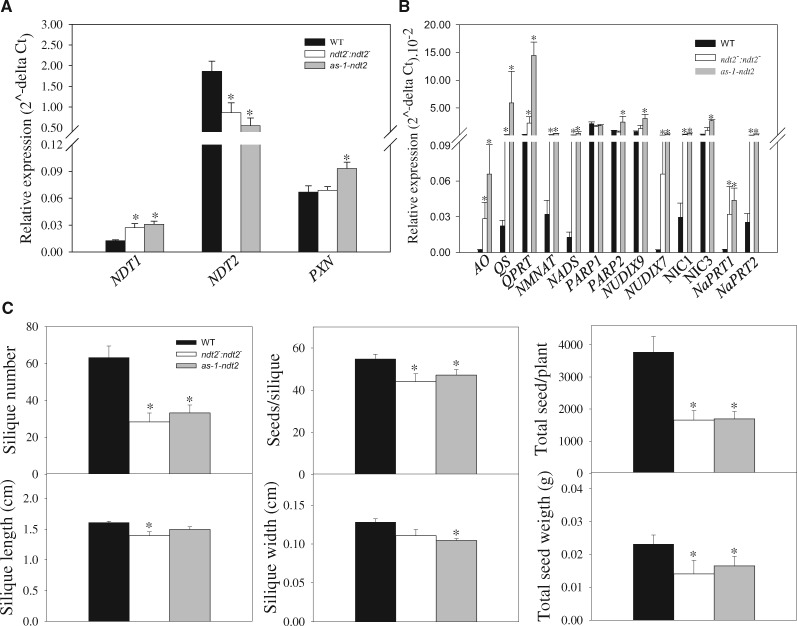
Gene expression analysis and evaluation of reproductive parameters in opened flowers of *NDT2* mutant lines and WT plants. (A) Expression of NAD^+^ transporters *NDT1*, *NDT2* and *PXN* genes in *ndt2^−^:ndt2^−^*, *as-1-ndt2* and WT. (B) Expression of NAD^+^ biosynthetic pathway genes: *AO*, *quinolinate synthase* (*QS*), *QPRT*, *NaMNAT*, *NAD synthetase* (*NADS*), *PARP*, *nucleoside diphosphates linked to some moiety X* (*NUDIX*), *nicotinate phosphoribosyltransferase* (*NaPRT*) and *NIC* in *NDT2* mutant lines. (C) Reproductive phase analysis of WT and *NDT2* mutant lines as the silique number, seeds/silique, total seed/plant, silique length, silique width and total seed weight. The values indicate means of four repeats � SE for gene expression and 12 replicates � SE for reproductive phase (indicated by error bars). Asterisks indicate values that were determined by Student’s *t*-test to be significantly different (*P *<* *0.05) from WT.

### Responses of reduction in *NDT2* expression on primary metabolism in seeds, flowers and siliques

Considering that genes belonging to the NAD^+^ biosynthetic pathway were upregulated in flowers of plants with reduced *NDT2* expression and that the reproductive phase in these plants was negatively affected, we next decided to evaluate the metabolite levels of seeds, imbibed seeds and germinated seeds, two days after stratification (DAS) in *ndt2*^*−*^*:ndt2*^*−*^ line and WT (Fig.�3). These studies revealed considerable changes in the levels of a wide range of amino acids, organic acids and sugars. In seeds, low expression of *NDT2* resulted in the accumulation of alanine, aspartate, arginine, glutamine, glycine, isoleucine, methionine, phenylalanine, serine, threonine and valine, whilst tyrosine was decreased. The amount of organic acids: aconitate, citrate, lactate, malate and shikimate were augmented, as well as sugars: fructose, fucose, glucose, galactose, isomaltose, mannose and trehalose. In the same manner, fructose-6-phosphate was increased. In imbibed seeds, the majority of detected metabolites decreased in the *ndt2*^*−*^*:ndt2*^*−*^ line in comparison to WT, including levels of the amino acids alanine, aspartate, asparagine, glutamine, glycine, methionine, serine, tyrosine and valine, the organic acids fumarate, glycerate and GABA, and the sugars: galactose and fructose-6-phosphate, and the polyols: erythritol, sorbitol, glycerol and threitol. By contrast, higher levels of citrate, malate, mannose and myo-inositol were detected in *ndt2*^*−*^*:ndt2* imbibed seeds in comparison to their WT counterparts. In germinated seeds from *ndt2*^*−*^*:ndt2*^*−*^ lines, the majority of amino acids, including alanine, asparagine, glutamate, glutamine, homoserine, isoleucine, leucine, lysine, methionine, phenylalanine, tyrosine and valine, displayed reduced levels. By contrast, the levels of aspartate, serine, threonine, ascorbate, aconitate, citrate, dehydroascorbate, glycerate, malate, fructose, fucose and mannose increased in the *ndt2*^*−*^*:ndt2*^*−*^-germinated seeds. We additionally evaluated the relative metabolite levels in immature flowers, opened flowers, immature and mature siliques of *ndt2*^*−*^*:ndt2*^*−*^ and WT plants (Fig.�3). The levels of amino acids, organic acids and sugars in immature flowers and opened flowers of the *ndt2*^*−*^*:ndt2*^*−*^ line did not show significant changes. By contrast, the levels of the amino acids: aspartate, glutamate and homoserine (also in siliques) were higher in immature siliques of *ndt2*^*−*^*:ndt2*^*−*^ lines, whilst the levels of ornithine were elevated in mutants in mature mutant siliques. Therefore, these results describe the importance of the correct *NDT2* expression during seed development and metabolism.

**Fig. 3:**
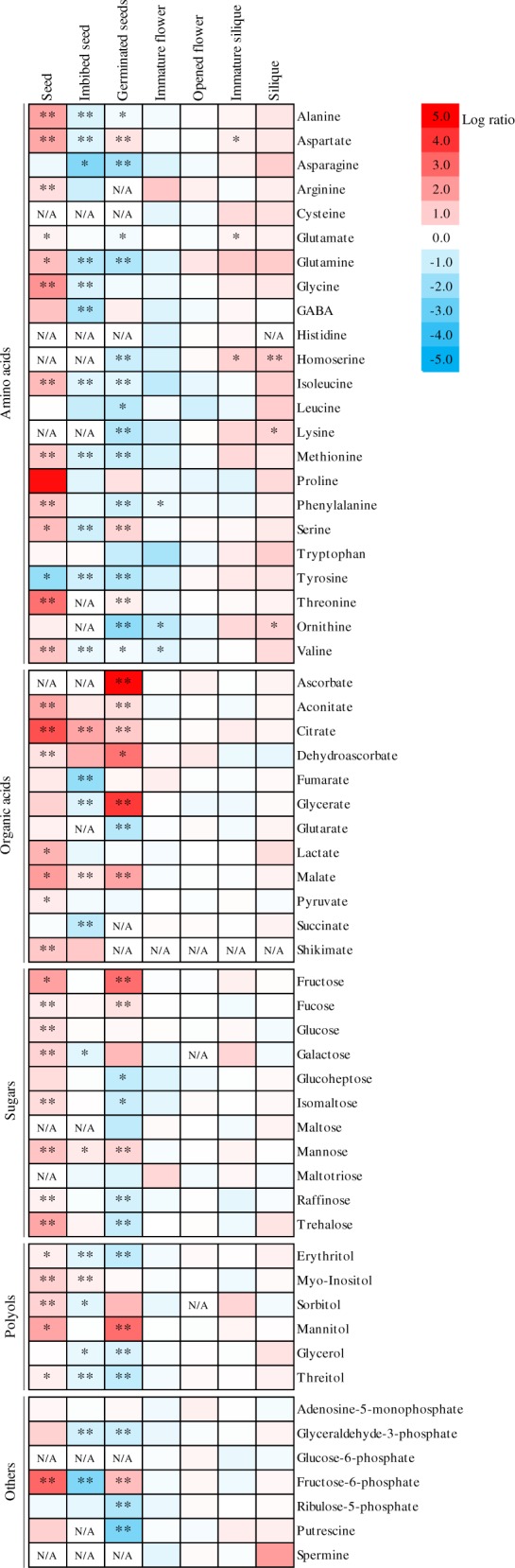
Heat map representing the changes in seed, inflorescence and silique metabolite contents of *ndt2^−^:ndt2^−^* mutants relative to the WT. Metabolite levels in mutants were normalized to the internal standard ribitol before normalization to the average abundance across all stages. Values represent average ratios of normalized metabolite levels and are expressed as log_2_ relative to WT in the corresponding tissue (*n* = 5): blue (low) to red (high) by the false color code as −5 < 0<5. Boxes labeled *N/A* indicate that metabolite ratios could not be determined, as the compounds were identified in the mutants but not in the WT, or vice versa. Asterisks denote significant differences of individual metabolites between mutants and the WT in the corresponding tissue according to the Student’s *t*-test with significant values *P <* *0.05 or **P <* *0.01.

### Consequences of reduced *NDT2* expression on heterotrophic tissues

Promoter analysis revealed that *NDT2* is highly expressed in developing tissues and in cells with high metabolic activity (Palmieri et�al. 2009). Furthermore, metabolite profiling showed strong differences between *ndt2* and WT plants in dry and imbibed seeds (Fig.�3). This urged us to examine seed germination and seedling development in *ndt2*^*−*^*:ndt2*^*−*^ and WT. We observed that seeds from *ndt2*^*−*^*:ndt2*^*−*^ plants displayed lower germination rates (Fig.�4A). Germination and seedling growth in the *ndt2*^*−*^*:ndt2*^*−*^ genotype were also considerably slower than in WT, as indicated by the decrease in germination speed index (GSI) and emergency speed index (ESI) values (Fig.�4B, C). Since the low *NDT2* expression altered germination and seedling development, we next evaluated the fatty acid profile of seeds and seedlings at 2, 4 and 6 DAS (Fig.�4D–F). When analyzing dry seeds, it was noticed that the fatty acids 16:0, 18:0, 20:0, 20:1a, 22:1 and 24:1 increased whilst fatty acids 17:0 and 18:2 diminished (Fig.�4D). Despite these changes, the total level of fatty acids was unaltered (Fig.�4D″). Two DAS, the seedlings from the *ndt2*^*−*^*:ndt2*^*−*^ line were unaltered from the WT both in terms of the individual fatty acid methyl esters (FAMEs) and the total fatty acid level (Fig.�4E′, E″). However, at 4 DAS, there was a decrease in the level of FAMEs of lower carbon number, such as 16:3, 16:1, 17:0, 18:3 and 18:2, and an increase in the levels of FAMEs with higher carbon number, such as 14:0, 18:1, 18:0, 20:0, 20:1a, 20:1b, 22:1 and 24:1 (Fig.�4F′). The total level of fatty acids was also increased at this time point (Fig.�4F″). By contrast, at 6 DAS, relatively minor changes in the FAME content were observed. Only the FAMEs 14:0 and 18:1 increased, whilst 16:3 and 16:1 decreased (Fig.�4G′), and similarly, the total fatty acid level was unaltered in comparison to the WT at this time point (Fig.�4G″). Considering the overall changes in the fatty acids profile and taking into account that the eicosenoic acid C20:1, which is considered the marker fatty acid in Arabidopsis (Chaves et�al. 2019), generally increased in the *NDT2* mutants, it might be suggested that the *ndt2*^*−*^*:ndt2*^*−*^ line has a compromised storage reserve mobilization.

**Fig. 4:**
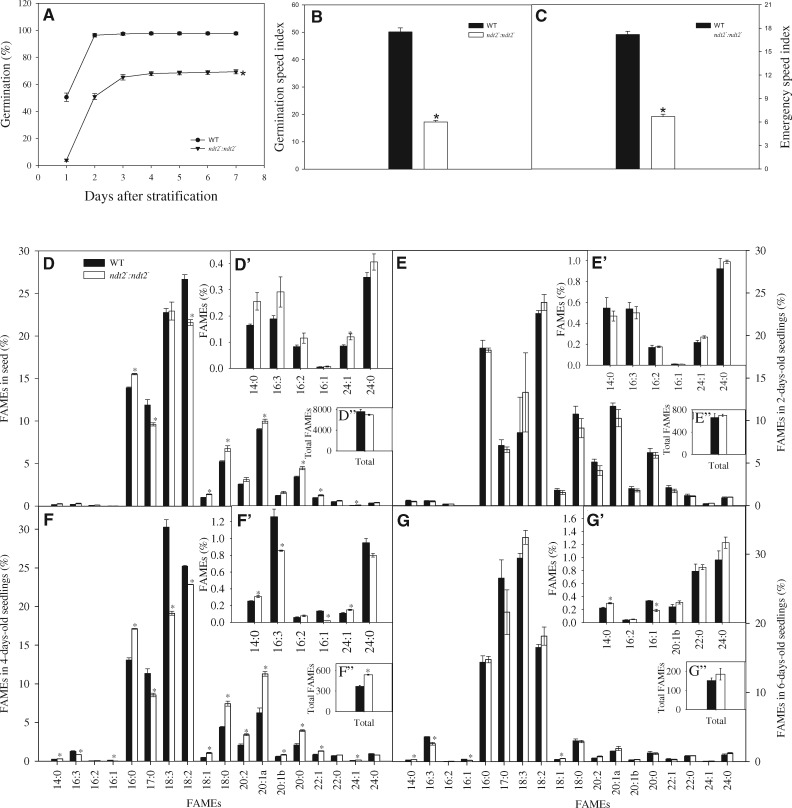
Seed germination and fatty acids content of plants with the reduced expression of *NDT2*. (A) Percentage of seed germination; (B) GSI; and (C) ESI and relative levels of FAMEs in seeds and seedlings with the reduced expression of *NDT2*. (D) Seeds, (E) 2 day-old seedlings, (F) 4 day-old seedlings and (G) 6 day-old seedlings. (D′–G′) Details presenting FAMEs for best visualization. (D″–G″) Mean of total of FAMEs per sample. Values in (A)–(C) are presented as mean � SE of determinations on six plates with 50 seeds each per line. Values in (D)–(G) are presented as mean � SE of determinations on three individual samples per line. Asterisks indicate values that were determined by Student’s *t*-test to be significantly different (*P *<* *0.05) from wild type (WT).

Considering the alterations in germination and seedling development of plants with low *NDT2* expression, we next evaluated root growth. For this purpose, WT, *ndt2*^*−*^*:ndt2*^*−*^ and *as-1-ndt2* lines were grown on Murashige and Skoog (MS)/agar plates with or without 1% (w/v) sucrose (Supplementary Fig. S1). Significant differences between WT and *ndt2*^*−*^*:ndt2*^*−*^ plants were observed in the presence of 1% sucrose, whereas *as-1-ndt2* line exhibited moderately higher root growth compared to WT (Supplementary Fig. S1). Similarly, on MS media without sucrose, root growth was higher for *ndt2*^*−*^*:ndt2*^*−*^ compared to WT root growth (Supplementary Fig. S1). Together, these results indicate that proper *NDT2* expression is required to regulate proper seed germination and seedling establishment.

### Effects of reduced *NDT2* expression on gene expression and pyridine nucleotide levels during seedling establishment

The reduced *NDT2* expression strongly impacted seed production, germination and seed metabolism (Figs.�2–4). Furthermore, plants with reduced *NDT2* expression were shown to have a higher root growth despite a slower ESI (Supplementary Fig. S2, Fig.�4B). Moreover, NAD^+^ carrier genes and genes encoding enzymes for NAD^+^ biosynthesis were upregulated in flowers of *ndt2* plants (Fig.�2A, B). To verify a possible link between these phenotypes with NAD^+^ transport and NAD^+^ metabolism, we next measured the expression of NAD^+^ carrier genes, genes encoding enzymes for NAD^+^ biosynthesis and the levels of pyridine nucleotides during seedling establishment. For gene expression analysis, total RNA was extracted from seedlings after 2, 4 and 8 DAS. No differences between *ndt2* and WT were observed for *NDT1* expression, whilst *PXN* expression was decreased in the mutant line in all evaluated time points (Fig.�5A). In addition, all the genes evaluated here encoding enzymes of the de novo and salvage pathways of NAD^+^ biosynthesis were upregulated in the *ndt2* line at 4 and 8 DAS, except for AO at 8 DAS (Fig.�5B). Subsequently, we determined the cellular NAD^+^, NADH, NADP^+^ and NADPH pools in whole seedlings harvested in the middle of the light period (Fig.�5C). The levels of NAD^+^ were shown to be increased in the mutant line at 4 DAS compared to the WT (Fig.�5C). Furthermore, the NADPH/NADP^+^ ratio was higher in *ndt2* compared to WT at 4 DAS, whilst no changes were observed in the levels of NADH, NADP^+^ and NADPH in the mutants in comparison to their WT counterparts. Together, these results highlight the importance of NAD^+^ transport and metabolism to regulate the expression of genes belonging to NAD^+^ biosynthesis and to adjust the NAD(P)(H) pool along with seed production, germination and seedling establishment in Arabidopsis.

**Fig. 5:**
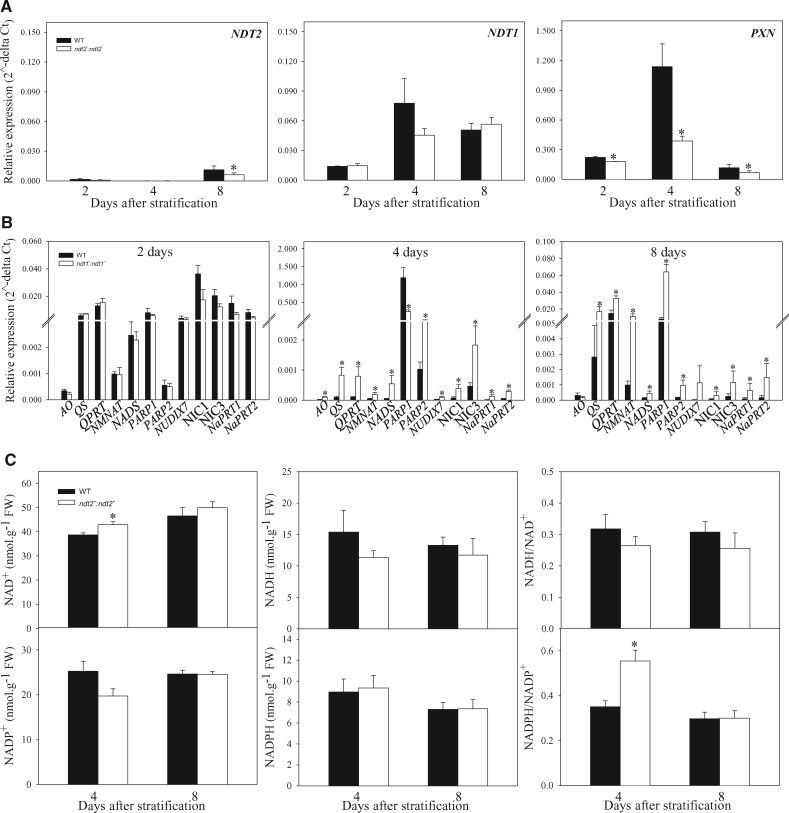
Gene expression analysis and changes in the pyridine nucleotide levels in seedlings of *NDT2* mutant lines and wild type (WT) plants. (A) Expression of NAD^+^ transporters *NDT2*, *NDT1* and *PXN* genes in *ndt2^−^:ndt2^−^* and WT. (B) Expression of NAD^+^ biosynthetic pathway genes: *AO*, *quinolinate synthase* (*QS*), *QPRT*, *NaMNAT*, *NAD synthetase* (*NADS*), *PARP*, *nucleoside diphosphates linked to some moiety X* (*NUDIX*), *nicotinate phosphoribosyltransferase* (*NaPRT*) and *NIC* in *NDT2* mutant and WT seedlings. (C) Changes in the pyridine nucleotides in 4- and 8-day-old seedlings harvested in the middle of the light period, NAD^+^, NADH, NADH/NAD^+^, NADP^+^, NADPH, NADPH/NADP^+^. The values indicate means of four repeats � SE for gene expression and five replicates � SE for pyridine nucleotides (indicated by error bars). Asterisks indicate values that were determined by Student’s *t*-test to be significantly different (*P *<* *0.05) from WT.

## Discussion

### The biochemical role of the NDT2 transporter in Arabidopsis

The recombinant NDT2 was demonstrated to be an NAD^+^ carrier in plant mitochondria (Palmieri et�al. 2009). Like the yeast carrier *Sc*NDT1 (Todisco et�al. 2006), *At*NDT2 has an affinity of 0.15 mM for NAD^+^, which is similar to the NAD^+^ affinity measured in isolated mitochondria of potato tubers (Tobin et�al. 1980). Given that the sum of NAD^+^ and NADH can reach concentrations of 0.64 mM in the cytosol of plant cells (Igamberdiev and Gardestr�m 2003), it was suggested that *At*NDT2 might allow a substantial influx into the mitochondria under *in vivo* conditions. Accordingly, this is in line with the significant reduction in the mitochondrial NAD^+^ level MOSAIC DEATH 1 (MOD1) suppressor line (SOM592), identified as an *ndt2* mutant by screening the ethyl methanesulfonate-mutagenized seeds of *mod1* Arabidopsis plants (Luo et�al. 2019). Measurements of pyridine nucleotide contents in isolated mitochondria of 4-week-old leaves of *som592*/*ndt2* mutants revealed that mitochondrial NAD^+^ level was reduced, whilst the mitochondrial NADH level exhibited no significant difference compared to WT levels, confirming that the mutation in *NDT2* leads to a decreased mitochondrial NAD^+^ uptake activity in Arabidopsis (Luo et�al. 2019).

Given that mitochondria contain a significant amount of NAD^+^, up to 2 mM in pea leaf protoplasts (Igamberdiev and Gardestr�m 2003), it would be anticipated that a reduction in *AtNDT2* expression would lead to severe consequences for Complex I and NAD(P)H dehydrogenase-dependent respiration. However, we must consider that *At*NDT1, until very recently presumed to exclusively reside in the chloroplast, has genuinely been demonstrated to localize in the mitochondrial membrane (Chaves et�al. 2019). The fact that the expression of *NDT1* was increased in flowers of *ndt2* plants (Fig.�2A) demonstrates that compromised NAD^+^ uptake caused by *At*NDT2 deficiency might be, at least partially, compensated by NDT1 activity. In addition, *At*NDT1 and *At*NDT2 have different kinetic constants, with *At*NDT2 exhibiting a higher affinity for NAD^+^ than *At*NDT1 (Palmieri et�al. 2009). The crossing of *mod1* with two T-DNA insertion mutants of *NDT1* revealed that none of *mod1ndt1* double mutants could rescue the *mod1* phenotype (Luo et�al. 2019). Thus, *At*NDT2 seems to be a key regulator of the mitochondrial NAD^+^ and NADH pools, and compromised NAD^+^ import activity in *ndt2* mutants cannot be fully compensated for by other transporters, highlighting the functional importance of NDT2 for NAD^+^ import by plant mitochondria.

### Low expression of *NDT2* impacts seed germination and seedling establishment

In silico expression analyses revealed that *NDT2* is highly expressed in mature seeds (Supplementary Fig. S2). We found that the *ndt2*^*−*^*:ndt2*^*−*^ mutant harbors both a reduction and a delay in germination, and increased abnormal seedlings production (Fig.�4A, B, Supplementary Fig. S1). Previous studies have revealed the influence of NAD^+^ in seed germination (Hunt et�al. 2007, Hunt and Gray 2009, Zeng et�al. 2014, Pham et�al. 2015, Chaves et�al. 2019). Mature seeds express the *NIC2* gene encoding nicotinamidase enzyme at relatively high levels (Hunt et�al. 2007, Hunt and Gray 2009). Mutant plants for this enzyme have lower and slower germination rates, followed by an increase in NAD^+^ content (Hunt et�al. 2007). In addition, mutants with reduced expression of *PARP1* and *PARP2* genes displayed increased NAD^+^ levels in leaves and decreased seed germination (Pham et�al. 2015). In addition, Arabidopsis *nudix hydrolase 7* (*At*nudt7) mutants exhibited reduced seed germination and excessive ABA accumulation (Zeng et�al. 2014). Recently, we showed the importance of the mitochondrial NAD^+^ transporter NDT1 for NAD^+^ homeostasis and seed germination (Chaves et�al. 2019). Together, these studies indicate that changes in NAD^+^ metabolism affect NAD^+^-dependent processes required during germination. Consistent with this suggestion is the observed correlation between total cellular NAD^+^ levels and deep seed dormancy (Hunt and Gray 2009). Furthermore, the seed germination process is associated with the presence of a mechanism that regulates the level of NAD^+^ or the NAD^+^/NADP^+^ ratio to adjust the balance between NADP^+^ biosynthesis and catabolism. Interestingly, in this work, seed dormancy and germination were linked to increased NAD^+^ levels possibly leading to ABA production (Hunt and Gray 2009). Accordingly, the levels of NAD^+^ were shown to be increased in the *ndt2*^*−*^*:ndt2*^*−*^ line after 4 d of light (Fig.�5C).

Mature dry seeds of the *ndt2*^*−*^*:ndt2*^*−*^ mutant line displayed increased levels of amino acids, organic acids and sugars compared to WT seeds (Fig.�3). The de novo NAD^+^ biosynthesis pathway in plants has aspartate as its precursor (Katoh et�al. 2006, Takahashi et�al. 2006, Schippers et�al. 2008). Interestingly, dry seeds of the *ndt2*^*−*^*:ndt2*^*−*^ mutant accumulate aspartate and its derivative amino acids, such as lysine, methionine, threonine and isoleucine (Fig.�3). Together with the upregulation of NAD^+^ biosynthetic genes in flowers (Fig.�2B) and the increased levels of NAD^+^ after 4 d of light (Fig.�5C), these results suggest that NAD^+^ might be accumulated during seed development and may be associated with the reduced and delayed seed germination observed in the *ndt2*^*−*^*:ndt2*^*−*^ mutant. In imbibed and germinated seeds, some of the amino acids subsequently decreased (Fig.�3). This might suggest that *ndt2*^*−*^*:ndt2*^*−*^ seeds failed to incorporate these metabolites into storage reserves during seed maturation and, thus, the lower and slower germination could also be associated with a significant increase in the levels of some sugars like glucose, fructose and mannose. Previous studies demonstrated that exogenous glucose may modulate internal ABA concentrations by increasing ABA synthesis or inhibiting its degradation, which results in a delay to germination and an inhibition of seedling development (Price et�al. 2003). In addition, it has been demonstrated that perturbation in NAD^+^/NADH homeostasis in seeds can alter ABA and reactive oxygen species levels in seeds, which in turn impairs germination (Zeng et�al. 2014). Changes in the levels of reduced and oxidized pyridine nucleotides suggested that breaking of dormancy is associated with a reduction in NAD^+^ levels but not with an increase in NADP^+^ levels (Hunt and Gray 2009). Therefore, the NAD^+^/NADP^+^ ratio may be involved in determining or controlling seed dormancy. Taken together, these results suggest that NDT2 activity is essential to maintain a proper NAD^+^ balance in cellular compartments in seeds and thus sustain metabolic adjustments required for seed maturation and germination, as well as for seedling establishment.

Changes in fatty acids levels occur during seedling formation in lines with reduced *NDT2* expression (Fig.�4). Increases in the relative contents of some fatty acids, especially eicosanoic acid (20:1), which is considered a triacylglycerol storage marker in *Arabidopsis thaliana* seeds (Lemieux 1990), corroborate to the delay in seedling formation, and this delay may be associated with β-oxidation. During β-oxidation, fatty acids are metabolized along with NADPH and ATP generation. Therefore, an accumulation of fatty acids suggests that β-oxidation was delayed in the *ndt2* plants. In accordance, *PXN* expression was shown to be reduced in *ndt2* plants during seedling development (Fig.�5A), suggesting that peroxisomal NAD^+^ content was affected. Reduced expression of the peroxisomal and mitochondrial NAD^+^ transporters, PXN (Bernhardt et�al. 2012) and NDT1 (Chaves et�al. 2019), respectively, resulted in similar changes in the fatty acid profile, suggesting that NAD^+^ import into organelles is likely vital in the process. However, further studies are necessary to unravel the interactions between β-oxidation and NAD^+^ transport. Collectively, these results suggest that a slight accumulation of certain fatty acids, coupled with changes in primary metabolism and an increase in NAD^+^ levels are likely responsible for the pattern of germination and seedling establishment phenotypes observed in plants with reduced *NDT2* expression.

### Reduced expression of *NDT2* negatively impacts the reproductive phase

The *NDT*2 gene is highly expressed in Arabidopsis pollen compared to seeds, leaves and flowers (Chaves et�al. 2019). The great *NDT2* expression in sexual reproductive tissues led us to investigate the importance of *NDT2* during the reproductive phase. *ndt2* plants were negatively affected in their reproductive phases (Fig.�2C). These plants displayed a reduced number of siliques per plant and a diminished number of seeds per silique, leading to reductions in total seed production (Fig.�2C). Recently, we have demonstrated the importance of *NDT1* in the regulation of seed production (Chaves et�al. 2019). Our results shown that *ndt1* mutants exhibited a reduced seed yield due to low pollen viability and low pollen tube growth because of dysfunctional NAD^+^ homeostasis in cellular subcompartments (Chaves et�al. 2019). In addition to the importance of NAD^+^ compartmentation, a number of studies have shown the role of NAD^+^ during seed formation (Chai et�al. 2005, Katoh et�al. 2006, Hashida et�al. 2009). The levels of NAD^+^ were shown to be much higher in flowers and siliques compared to seedlings, roots and leaves, which is in accordance with the previous demonstration that the NAD(P)(H) pool is important in the reproductive phase (Wang and Pichersky 2007). In addition, the embryo lethal phenotype observed for AO, QS and QPT mutant lines points again to the pivotal importance of NAD^+^ biosynthesis for seed production (Katoh et�al. 2006). Furthermore, mutants for the *NADK2* gene displayed reduced silique sizes and reduced seed production (Chai et�al. 2005). Moreover, a direct correlation between NAD^+^ levels and pollen tube growth has been studied (C�rdenas et al. 2006, Hashida et�al. 2007, Hashida et�al. 2013a, Hashida et�al. 2013b). Since pollens with lower NAD^+^ levels prematurely germinate and considering the observed decrease in NAD^+^ content during pollen tube growth (Hashida et�al. 2013a, Hashida et�al. 2013b), it is assumed that NAD^+^ content negatively regulates pollen germination. Indeed, Hashida et�al. (2007) showed a high nicotinate/nicotinamide mononucleotide adenyltransferase (NMNAT) activity during pollen development, which led to high levels of NAD^+^ biosynthesis. Therefore, the *NMNAT* mutant presented decreased NAD^+^ levels, consequently having altered pollen tube germination (Hashida et�al. 2007). Here, we also demonstrate that NAD^+^ biosynthesis-related genes were upregulated in flowers of *ndt2* plants (Fig.�2B), suggesting an attempt to maintain the NAD^+^ homeostasis required in flowers to properly produce seeds. These results confirm the importance of the correct NAD^+^ uptake into mitochondria by NDT2 on the regulation of seed production in Arabidopsis.

### Downregulation of *NDT2* expression impacts the *de novo* and salvage pathways of NAD^+^ biosynthesis

Given that the pools of pyridine nucleotides in different compartments are linked by NAD^+^ transporters, we hypothesized that the impairment of NAD^+^ import by NDT2 in mitochondria would potentially impact on the activity of NDT1 and PXN transporters to balance intracellular NAD^+^ levels. Accordingly, expression of both *NDT1* and *PXN* was increased in *ndt2* flowers (Fig.�2A). However, *PXN* expression was shown to be reduced during seedling establishment in *ndt2* plants (Fig.�5A). This suggests that the deficiency of NAD^+^ uptake by the mitochondria in the *NDT2* mutants differentially affects the expression of the other NAD^+^ carrier, depending on the organ and developmental stage.

There is a direct link between the manipulation of genes encoding enzymes of NAD^+^ biosynthetic pathways and changes in the NAD^+^ levels (Hunt et�al. 2007, Wang and Pichersky 2007, Schippers et�al. 2008). For instance, *ndt1* mutants also displayed differential expression of genes related to NAD^+^ biosynthesis; *QPRT* and *NMNAT* showed higher expression in imbibed seeds, whilst *QS* expression was reduced in *ndt1* leaves (Chaves et�al. 2019). Moreover, the reduced expression of *QS* increased the NAD^+^ content along with increased transcript levels of all genes from the de novo and salvage pathways of NAD^+^ biosynthesis (Schippers et�al. 2008). Similarly, the expression of *NIC1*, a nicotinamidase encoding gene, was positively correlated with NAD^+^ levels in Arabidopsis (Hunt et�al. 2007, Wang and Pichersky 2007). Furthermore, the downregulation of *PARP1* and *PARP2* genes positively increased NAD^+^ levels (Pham et�al. 2015). In addition, it has been described that upregulation of the salvage pathway occurs under conditions of oxidative stress (Schippers et�al. 2008). We here demonstrated that reduced *NDT2* expression increased the expression of genes encoding enzymes from the de novo and salvage NAD^+^ biosynthesis pathway in flowers and during seedling establishment (Figs.�2B, 5B). Some possibilities could explain the increased gene expression: (i) the reduced import of NAD^+^ by mitochondria may itself generate a signal that leads to increments in the expression of NAD^+^ biosynthetic genes; (ii) the unbalanced levels of NAD^+^ due to the reduced *NDT2* expression may lead to misregulation of the NAD^+^ biosynthetic genes; or (iii) to maintain redox homeostasis, NAD^+^ and its derivatives may indirectly result in the upregulation of these genes. Although the transcriptional control of genes encoding enzymes for NAD^+^ biosynthesis in plants is still a mystery, the differential expression of NAD^+^ biosynthetic genes in *ndt2* mutant plants demonstrates, irrespective of which of these hypotheses is correct, that correct functional NAD^+^ mitochondrial import is sensed and NAD^+^ biosynthesis is upregulated when this import is constrained.

## Materials and Methods

### Isolation of the *ndt2* mutant line

The *ndt2*^*−*^*:ndt2*^*−*^ line was derived from the GABI-KAT collection (GK-143G09) and has a T-DNA insertion in the fifth exon, as well as a sulfadiazine selection marker gene (Fig.�1A). Progeny resulting from the selfing of this line were selected based on their antibiotic resistance, and homozygous plants were identified by PCR screening using specific primers for the *NDT2* gene and the T-DNA insertion (forward 5′-ATATTGACCATCATACTATTGC-3′, reverse 5′-TGGATCGCGACATGGCTTACTC-3′ and T-DNA 5′-ATATTGACCATCATACTCATTGC-3′) (Supplementary Fig. S3).

### Generation of *ndt2* antisense line

Transgenic lines with suppressed expression of the *NDT2* gene were generated by expressing the antisense cDNA fragment of 1,092 bp under the control of the *35S* promoter. Specific primers were designed (forward 5′-ATGATTGAACATGGGAACTCTACCTTTG-3′ and reverse 5′-TTATTTGCTTCCAAGAGGGATATGGG-3′) for the PCR amplification of full-length coding sequence of *At*1g25380 gene from a cDNA library derived from *A*. *thaliana* flowers. The purified PCR fragment was cloned into pDONR207 vector using the pDONR207 directional cloning kit (Thermo Fischer Scientific). The resultant ENTRY vector was used in the Gateway LR reaction (Thermo Fischer Scientific) with pH2WG7 Destination vector (Karimi et�al. 2002) for the cloning of the *NDT2* coding sequence in antisense orientation in the expression vector. *Agrobacterium tumefaciens* GV3101 was transformed with the expression vector and used for the transformation of *A*. *thaliana* ecotype Columbia-0 by floral dip (Clough and Bent 1998). The cassette also contained a hygromycin resistance marker gene with *nos* promoter and *nos* terminator (Fig.�1A). Initial screening of the 20 transgenic lines was carried out using a combination of hygromycin resistance and mRNA measurements. These screens allowed the selection of an antisense line, here named *as-1-ndt2*, with reduced expression of *NDT2*, which was used for subsequent physiological characterization.

### Plant material and growth conditions

Seeds of all genotypes were surface sterilized and germinated on half-strength MS medium (Murashige and Skoog 1962) supplemented with 1% (w/v) sucrose and the respective selective agent (sulfadiazine (Sigma-Aldrich) 5.25 mg l^*−*^^1^ or hygromycin (Sigma-Aldrich) 10 mg l^*−*^^1^ according to the genotype). After 4 d of stratification at 4�C, plates with seeds were taken into a growth chamber at 22 � 2�C, with 60% relative humidity, 150 �mol photons m^*−*^^2^ s^*−*^^1^ irradiance and 8 h of light/16 h of dark photoperiod for 10 d. After this period, the plantlets were transferred to 0.08-dm^3^ vessels containing commercial substrate Tropstrato HT^�^ and maintained under the same condition until the end of the reproductive phase. At this time, silique samples were snap-frozen in liquid nitrogen for subsequent biochemical analysis.

For germination analyses and determination of fatty acid profiles, the seeds were sterilized and germinated as described above and maintained in an air-conditioned growth room at 22 � 2�C, with 60% relative humidity, 150 �mol photons m^*−*^^2^ s^*−*^^1^ irradiance and 12 h of light/ 12 h of dark photoperiod.

### Gene expression analysis

Gene expression analysis was performed by quantitative real-time PCR (qRT-PCR). Total RNA was extracted from flowers using TRIzol^�^ Reagent (Ambion, Life Technologies, Carlsbad, CA, USA) according to the manufacturer’s instructions. Total RNA was quantified by spectrophotometer at 260 nm and then subjected to treatment with RNase-free DNase (Invitrogen™, Life Technologies Carlsbad, CA, USA). Approximately 2 �g of isolated RNA was used to synthesize the cDNA using the Improm-II™ Reverse Transcription System (Promega, Madison, WI, USA) and Oligo(dT)_15_, following the manufacturer’s recommendations. qRT-PCRs were performed using a 7300 Real-Time PCR System (Applied Biosystems, Foster, CA, USA) and Power SYBR^�^ Green PCR Master Mix (Life Technologies/Applied Biosystem, Foster, CA, USA). We used the *At*NDT2 gene-specific primers (forward 5′-CGATGCCATGTTCCAACTAC-3′ and reverse 5′-CATCAAAAGGGCCAAAAAGT-3′) and specific primers for *Actin2* gene as endogenous control for normalization purposes. Flowers from six plants were collected and pooled, and the qRT-PCR analysis was performed in duplicate by the following steps: 94�C for 10 min, 40 cycles of 94�C for 15 s, 58�C for 15 s and 72�C for 15 s. qRT-PCR analysis was also performed in seedlings at 2, 4 and 8 d after germination to verify the expression of genes encoding other NAD^+^ transporters and genes related to NAD^+^ biosynthesis and recycling (Supplementary Table S1).

### Morphological analysis

Reproductive parameters were analyzed at the end of the life cycle from 12 plants per genotype. Seven siliques per plant were photographed under a stereomicroscope, and the width, length and number of seeds/silique were measured. The total seed production per genotype was expressed as seed weight in grams from 12 independent plants per genotype.

### Biochemical analysis

Dry seeds, imbibed seeds, germinated seeds, immature flowers, open flowers, immature siliques and mature siliques were harvested and immediately snap-frozen in liquid nitrogen. The samples were then stored at −80�C until further analyses. Metabolite profiling was performed by gas chromatography–mass spectrometry method according to the protocol described by Lisec et�al. (2006). Metabolites were manually annotated, and ion intensity was determined using the reference library mass spectra and retention indices from the Golm Metabolome Database (http://gmd.mpimp-golm.mpg.de, Kopka et�al. 2005) and following the recommended reporting format for metabolic profiling (Fernie et�al. 2011). Aliquots of ∼25 mg of leaf samples were collected in the middle of the light period from seedlings at 4 and 8 DAS for the quantification of NAD^+^, NADH, NADP^+^ and NADPH according to the protocol described by Schippers et�al. (2008). The complete information of relative metabolite content is listed in Supplementary Table S2.

### Seed germination, seedling establishment and root growth

Seeds from homozygous T-DNA insertion line *ndt2*^*−*^*:ndt2*^*−*^ and WT plants were surface sterilized and germinated as described above. The proportion of seeds germinating, GSI, percentage of normal and abnormal seedlings (including pale seedlings) and ESI were evaluated. The GSI and ESI were calculated by the sum of the number of germinated seeds (or normal seedlings) every day, divided by the number of days elapsed between sowing and germination, according to Maguire (1962). Each determination was evaluated based on six replicates consisting of 50 seeds each. For root growth assay, seeds of WT, *ndt2*^*−*^*:ndt2*^*−*^ and *as-1-ndt2* were surface sterilized, subsequently sown on half-strength MS plates containing 0.8% agar and 0 or 1% sucrose and then grown vertically at 22�C with a photoperiod of 12 h light/12 h dark. The root growth was measured for 11 d to determine the rate of root growth per day.

### Fatty acid profiling

Seed and seedling fatty acids were extracted exactly as described previously (Focks and Benning 1998). For quantification by gas chromatography, pentadecanoic acid was used as an internal standard (Browse et�al. 1985).

### Statistical analysis

All data were obtained from experiments using a completely randomized design. Student’s *t*-test was performed using the algorithm embedded into Microsoft Excel (Microsoft, Seattle, WA, USA) with an alpha of 0.5.

## Funding

Conselho Nacional de Desenvolvimento Cient�fico e Tecnol�gico (CNPq) (484675/2013-3 to A.N.-N.); Funda��o de Amparo � Pesquisa do Estado de Minas Gerais (FAPEMIG) and Max Planck Society (to A.N.-N. and W.L.A.); Deutsche Forschungsgemeinschaft in the framework of the Transregional Collaborative Research Centre (TRR175 to H.E.N. and A.R.F.); CNPq (306818/2016-7 to A.N.-N. and 307979/2019-9 to W.L.A.); Coordena��o de Aperfei�oamento de Pessoal de N�vel Superior (CAPES) (to E.F.-A.); FAPEMIG (BDS-00003-14 to I.d.S.C.).

## Supplementary Material

pcaa017_Supplementary_Data
