# Supplementary material for: Downregulation of a Mitochondrial NAD+ Transporter (NDT2) Alters Seed Production and Germination in Arabidopsis
Source: Plant Cell Physiol. 2020 Feb 17;61(5):897–908. doi: 10.1093/pcp/pcaa017 (PMC7217668; doi:10.1093/pcp/pcaa017)
Supplement: pcaa017_Supplementary_Data [file pcaa017_supplementary_data.zip › pcaa017-suppl_data/pcp-2019-e-00571-File007.pdf]

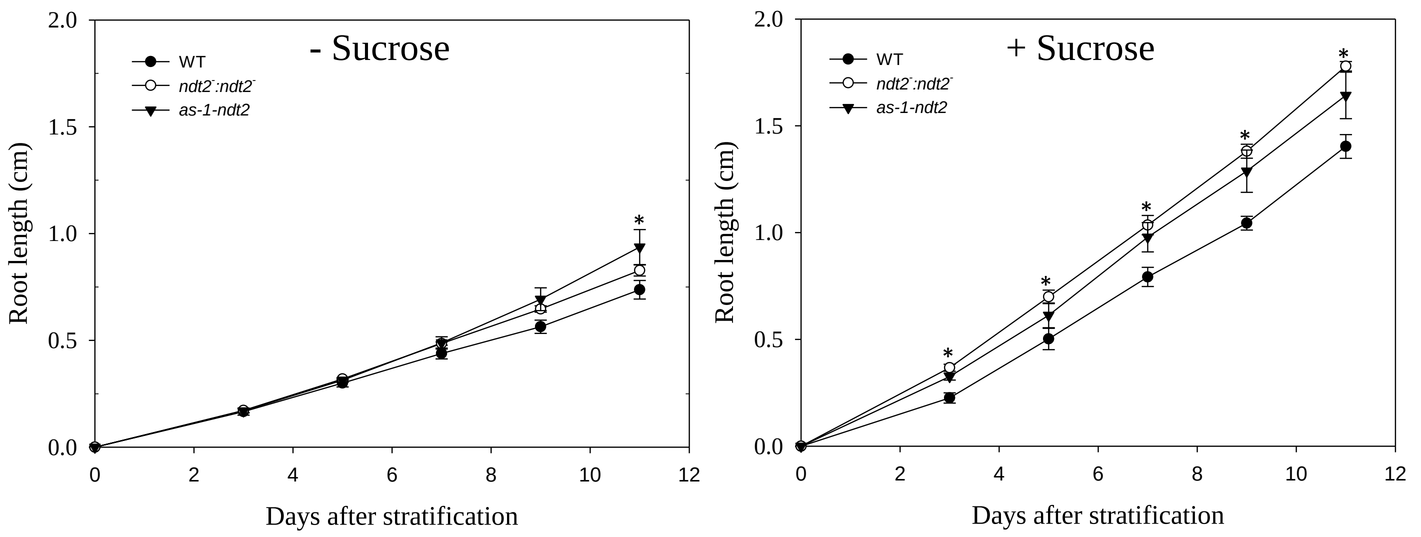

Supplementary figure S1 – Root growth of plants with reduced *NDT2* expression. Root growth in vertical plates in medium without and with 1% sucrose. Values are presented as mean ± SE of 4 individual plates. Asterisks indicate values that were determined by Student’s t-test to be significantly different ($P < 0.05$) from WT.
