# Supplementary material for: Downregulation of a Mitochondrial NAD+ Transporter (NDT2) Alters Seed Production and Germination in Arabidopsis
Source: Plant Cell Physiol. 2020 Feb 17;61(5):897–908. doi: 10.1093/pcp/pcaa017 (PMC7217668; doi:10.1093/pcp/pcaa017)
Supplement: pcaa017_Supplementary_Data [file pcaa017_supplementary_data.zip › pcaa017-suppl_data/pcp-2019-e-00571-File008.pdf]

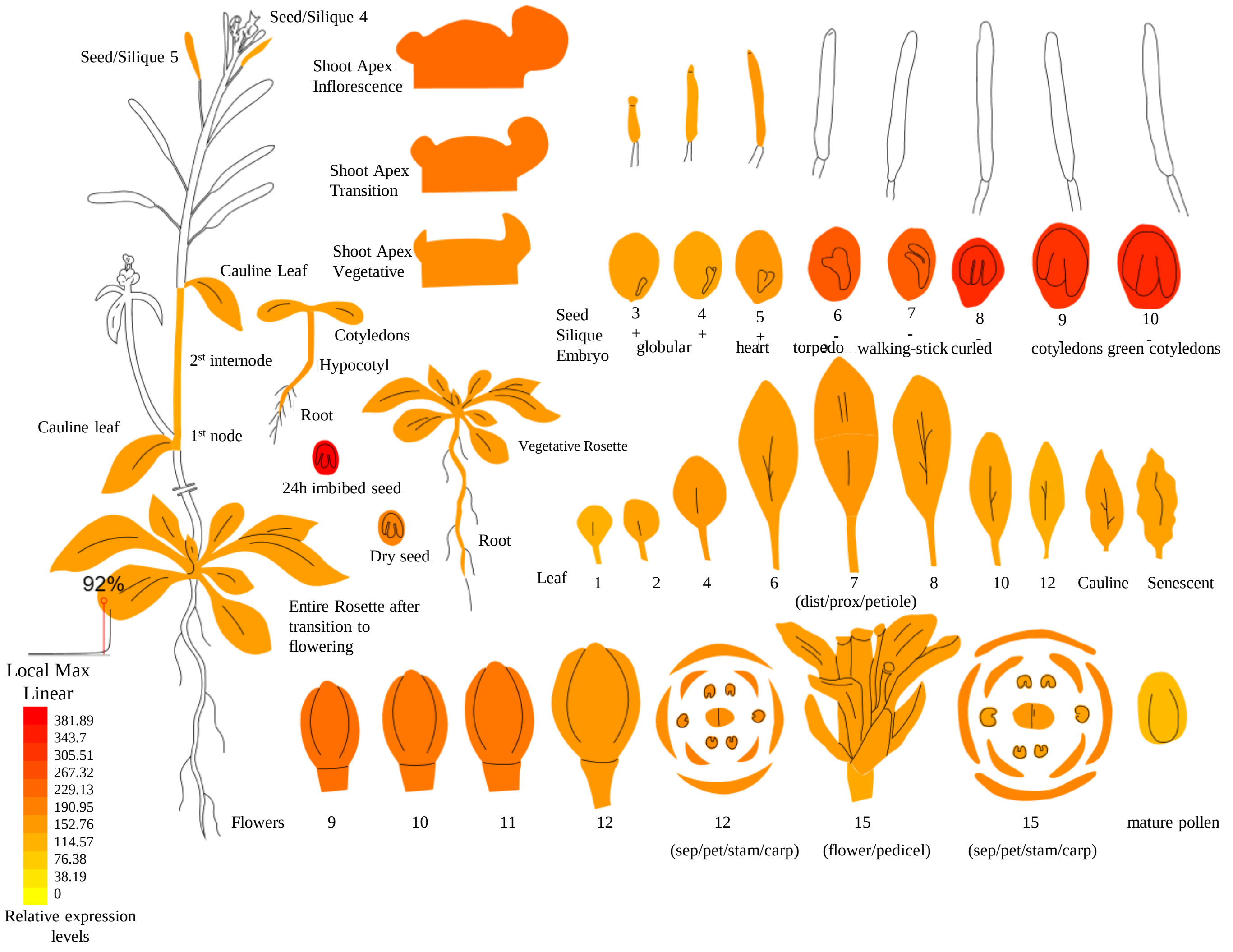

Supplementary figure S2. *In silico* analysis of the gene *NDT2* in different tissues.
Source: *A. thaliana* eFP Browser <http:// esc4037-shemp.csb.utoronto.ca/efp/cgi-bin/efp Web.cgi>.
