# Supplementary material for: Downregulation of a Mitochondrial NAD+ Transporter (NDT2) Alters Seed Production and Germination in Arabidopsis
Source: Plant Cell Physiol. 2020 Feb 17;61(5):897–908. doi: 10.1093/pcp/pcaa017 (PMC7217668; doi:10.1093/pcp/pcaa017)
Supplement: pcaa017_Supplementary_Data [file pcaa017_supplementary_data.zip › pcaa017-suppl_data/pcp-2019-e-00571-File010.pdf]

Supplementary Table S1. Genes and primer sequences used for the determination of the expression levels of genes related to NAD^+ metabolism and transport by qRT-PCR.

Gene code	Name	Foward	Reverse
*At*2g39970	PXN (Peroxissomal NAD transporter)	ACAACGTTACCGCTTTGGAGAC	TGACTGTAGCTCCGAGTTTCGC
*At*1g25380	NDT2 (mitochondrial NAD transporter)	CGATGCCATGTTCCAACTAC	CATCAAAAGGGCCAAAAAGT
*At*2g47490	NDT1 (Chloroplastic NAD transporter)	GGGAATTCGCGGATTGTACAGTGG	TGGGAAACTGAATGGCAACATGAC
*At*5g14760	Ao (Aspartate oxidase)	TGGTCGCTGGTGCTCATCTTTG	AGGCCCTTCAGTACACACAACTC
*At*5g50210	Qs (Quinolinate synthase)	TAGCAGGTGGTGAAGGTTGCTC	AGCGAGCTAAGCGAGTTCATCTTC
*At*2g01350	QPT (Quinolinic acid phosphoribosyl transferase)	TTGGGAAAGTATCAGGGAATGCAC	TGCAGCATCTGCCATTAACTTGG
*At*5g55810	NMNAT (Nicotinate/nicotinamide mononucleotide adenyltransferase)	TGGCAACTGGGAGTTTCAATCCTC	TCTCTCGCCAGCTCAAACATGC
*At*1g55090	NADS (NAD synthetase)	CAACAGCTGAGCTTGAGCCCATTC	CCATGTCGACTTCATCGAGCTGAG
*At*4g36940	NAPRT1 (Nicotinate phosphoribosyltransferase 1)	AGAACGAACCACCTCCAAAGGTC	AGCTCTTCCCTAGCTTCATCTGC
*At*2g23420	NAPRT2 (Nicotinate phosphoribosyltransferase 2)	AGTGCCACAACGTGTCGAAGAG	TTCTCTTGCTTCATCTGCACTTCC
*At*2g22570	NIC1 (Nicotinamidase1)	CGGCAATATGGCTCCAACAAAGC	GCAAGCTTTGCACTTTCCTCCAC
*At*5g23220	NIC3 (Nicotinamidase3)	ACTCGTCATCGACATGCAGAAC	CCTTTCACTTGCGTCACAGCAC
*At*4g12720	NUDIX7 (Nudix hydrolase 7)	AATTCTCTCCAAGGTACACACAGC	CCATAGGTTCCACCATGGTTACAG
*At*3g46200	NUDIX9 (Nudix hydrolase 9)	AGCTCCACACTGTCTCATTGGATG	TGATGGCAACCTGGCATTCTCG
*At*2g31320	PARP1 (Poly(ADP-ribose)polymerase1)	ATCGTCTACGATACAGCCCAGGTG	TGGTTCAGGCTCATCTCTTGTGC
*At*4g02390	PARP2 (Poly(ADP-ribose)polymerase2)	ATGCTACTCTGGCACGGTTCAC	AGGAGGAGCTATTCGCAGACCTTG
*At*2g37620	Actin	CTTGCACCAAGCAGCATGAA	CCGATCCAGACACTGTACTTCCTT
