# Supplementary material for: Downregulation of a Mitochondrial NAD+ Transporter (NDT2) Alters Seed Production and Germination in Arabidopsis
Source: Plant Cell Physiol. 2020 Feb 17;61(5):897–908. doi: 10.1093/pcp/pcaa017 (PMC7217668; doi:10.1093/pcp/pcaa017)
Supplement: pcaa017_Supplementary_Data [file pcaa017_supplementary_data.zip › pcaa017-suppl_data/pcp-2019-e-00571-File011.pdf]

Supplementary Table S2 A. Relative metabolite content in dried seeds, imbibed and germinated seeds of WT and *ndt2-:ndt2-* plants. Metabolite amount was normalized to the internal standard ribitol before normalization to the average abundance across all stages. Values represent average ratios of normalized metabolite levels between mutant and the wild type in the corresponding tissue. Data are presented as means ± SD (n=5). Values set in bold were determined by the Student's *t* test to be significantly different ($P < 0.05$) from their corresponding WT.

	Dried seeds		Imbibed seeds		Germinated seeds	
**Amino acids**	WT	*ndt2-:ndt2-*	WT	*ndt2-:ndt2-*	WT	*ndt2-:ndt2-*
Alanine	1.00 ± 0.000	**1.83 ± 0.000**	1.00 ± 0.000	-0.70 ± 0.000	1.00 ± 0.000	**-0.23 ± 0.000**
Aspartate	1.00 ± 0.006	**1.72 ± 0.014**	1.00 ± 0.002	-0.61 ± 0.002	1.00 ± 0.004	**0.53 ± 0.001**
Asparagine	1.00 ± 0.026	-0.33 ± 0.007	1.00 ± 0.013	-2.43 ± 0.001	1.00 ± 0.009	**-1.79 ± 0.004**
Arginine	1.00 ± 0.000	**0.63 ± 0.000**	1.00 ± 0.000	-0.98 ± 0.000	1.00 ± 0.000	N/A
Cysteine	1.00 ± 0.000	N/A	1.00 ± 0.000	N/A	1.00 ± 0.000	N/A
Glutamate	1.00 ± 0.070	**0.27 ± 0.093**	1.00 ± 0.044	-0.11 ± 0.015	1.00 ± 0.066	**-0.20 ± 0.145**
Glutamine	1.00 ± 0.000	**1.27 ± 0.000**	1.00 ± 0.000	**-1.46 ± 0.000**	1.00 ± 0.003	**-1.53 ± 0.002**
Glycine	1.00 ± 0.001	**2.14 ± 0.002**	1.00 ± 0.001	**-0.73 ± 0.001**	1.00 ± 0.002	-0.25 ± 0.000
Histidine	1.00 ± 0.000	N/A	1.00 ± 0.000	N/A	1.00 ± 0.000	N/A
Homoserine	1.00 ± 0.000	N/A	1.00 ± 0.000	N/A	1.00 ± 0.000	**-1.04 ± 0.001**
Isoleucine	1.00 ± 0.004	**1.36 ± 0.009**	1.00 ± 0.002	**-0.59 ± 0.002**	1.00 ± 0.029	**-0.55 ± 0.011**
Leucine	1.00 ± 0.002	0.03 ± 0.002	1.00 ± 0.002	-1.09 ± 0.001	1.00 ± 0.001	**-1.31 ± 0.000**
Lysine	1.00 ± 0.000	N/A	1.00 ± 0.000	N/A	1.00 ± 0.002	**-1.47 ± 0.001**
Methionine	1.00 ± 0.001	**1.05 ± 0.001**	1.00 ± 0.001	**-0.75 ± 0.001**	1.00 ± 0.003	**-1.05 ± 0.001**
Proline	1.00 ± 0.017	4.77 ± 0.399	1.00 ± 0.019	-0.58 ± 0.014	1.00 ± 0.098	0.60 ± 0.077
Phenylalanine	1.00 ± 0.001	**1.16 ± 0.001**	1.00 ± 0.001	-0.37 ± 0.000	1.00 ± 0.001	**-0.92 ± 0.000**
Serine	1.00 ± 0.003	**1.30 ± 0.007**	1.00 ± 0.003	**-0.92 ± 0.002**	1.00 ± 0.011	**0.81 ± 0.011**
Tryptophan	1.00 ± 0.001	0.17 ± 0.001	1.00 ± 0.000	0.08 ± 0.000	1.00 ± 0.003	-1.14 ± 0.001
Tyrosine	1.00 ± 0.001	**-2.08 ± 0.002**	1.00 ± 0.001	**-0.93 ± 0.001**	1.00 ± 0.009	**-1.48 ± 0.005**
Threonine	1.00 ± 0.000	**2.78 ± 0.000**	1.00 ± 0.000	**N/A**	1.00 ± 0.000	**0.31 ± 0.000**
Valine	1.00 ± 0.010	**1.14 ± 0.020**	1.00 ± 0.006	**-0.39 ± 0.006**	1.00 ± 0.034	**-0.22 ± 0.021**

Suplemental table . *(Continued)*

Organic Acids

Ascorbate	1.00 ± 0.000	N/A	1.00 ± 0.001	N/A	1.00 ± 0.001	**4.87 ± 0.004**
Aconitate	1.00 ± 0.000	**1.72 ± 0.000**	1.00 ± 0.000	0.43 ± 0.000	1.00 ± 0.000	**0.66 ± 0.000**
Citrate	1.00 ± 0.003	**3.44 ± 0.014**	1.00 ± 0.003	**1.78 ± 0.012**	1.00 ± 0.007	**1.05 ± 0.023**
Dehydroascorbate	1.00 ± 0.000	**0.57 ± 0.000**	1.00 ± 0.000	1.56 ± 0.000	1.00 ± 0.000	**2.75 ± 0.003**
Fumarate	1.00 ± 0.003	0.45 ± 0.024	1.00 ± 0.001	**-2.08 ± 0.001**	1.00 ± 0.001	0.19 ± 0.003
Glycerate	1.00 ± 0.001	0.89 ± 0.003	1.00 ± 0.000	**-0.66 ± 0.000**	1.00 ± 0.000	**3.91 ± 0.006**
Glutarate	1.00 ± 0.000	0.29 ± 0.000	1.00 ± 0.000	N/A	1.00 ± 0.000	**-1.38 ± 0.000**
GABA	1.00 ± 0.001	1.20 ± 0.006	1.00 ± 0.001	**-1.60 ± 0.000**	1.00 ± 0.021	0.34 ± 0.026
Lactate	1.00 ± 0.022	**1.48 ± 0.108**	1.00 ± 0.089	-0.41 ± 0.025	1.00 ± 0.020	-0.11 ± 0.014
Malate	1.00 ± 0.001	**1.94 ± 0.035**	1.00 ± 0.001	**0.42 ± 0.001**	1.00 ± 0.001	**1.78 ± 0.007**
Pyruvate	1.00 ± 0.000	**0.41 ± 0.000**	1.00 ± 0.000	-0.20 ± 0.000	1.00 ± 0.000	-0.27 ± 0.000
Succinate	1.00 ± 0.001	-0.11 ± 0.001	1.00 ± 0.000	**-1.24 ± 0.000**	1.00 ± 0.000	N/A
Shikimate	1.00 ± 0.000	**1.06 ± 0.000**	1.00 ± 0.000	1.07 ± 0.000	1.00 ± 0.000	N/A

Sugars

Fructose	1.00 ± 0.002	**1.86 ± 0.054**	1.00 ± 0.002	-0.03 ± 0.002	1.00 ± 0.005	**2.84 ± 0.025**
Fucose	1.00 ± 0.001	**0.40 ± 0.001**	1.00 ± 0.000	0.14 ± 0.000	1.00 ± 0.001	**0.58 ± 0.000**
Glucose	1.00 ± 0.005	**0.64 ± 0.022**	1.00 ± 0.050	0.06 ± 0.039	1.00 ± 0.018	0.14 ± 0.040
Galactose	1.00 ± 0.004	**0.88 ± 0.006**	1.00 ± 0.002	**-0.56 ± 0.000**	1.00 ± 0.000	1.37 ± 0.002
Glucoheptose	1.00 ± 0.024	0.67 ± 0.017	1.00 ± 0.004	-0.06 ± 0.004	1.00 ± 0.046	**-1.20 ± 0.021**
Isomaltose	1.00 ± 0.000	**0.69 ± 0.000**	1.00 ± 0.000	0.05 ± 0.000	1.00 ± 0.000	**-1.03 ± 0.000**
Maltose	1.00 ± 0.000	N/A	1.00 ± 0.000	N/A	1.00 ± 0.000	-1.22 ± 0.000
Mannose	1.00 ± 0.000	**1.17 ± 0.000**	1.00 ± 0.000	**0.43 ± 0.000**	1.00 ± 0.000	**0.81 ± 0.000**
Maltotriose	1.00 ± 0.005	N/A	1.00 ± 0.002	-0.33 ± 0.005	1.00 ± 0.005	-0.68 ± 0.003
Raffinose	1.00 ± 0.019	**0.28 ± 0.019**	1.00 ± 0.018	-0.13 ± 0.020	1.00 ± 0.004	**-0.81 ± 0.005**
Trehalose, alpha,alpha	1.00 ± 0.001	**1.75 ± 0.000**	1.00 ± 0.000	0.24 ± 0.000	1.00 ± 0.002	**-1.11 ± 0.001**

Suplemental table . *(Continued)*

**Polyols**						
Erythritol	1.00 ± 0.001	**0.32 ± 0.001**	1.00 ± 0.000	**-0.55 ± 0.000**	1.00 ± 0.000	**-1.20 ± 0.000**
Inositol, myo	1.00 ± 0.005	**0.91 ± 0.016**	1.00 ± 0.002	**0.33 ± 0.004**	1.00 ± 0.009	0.12 ± 0.008
Sorbitol	1.00 ± 0.004	**0.88 ± 0.006**	1.00 ± 0.002	**-0.56 ± 0.000**	1.00 ± 0.000	1.37 ± 0.002
Mannitol	1.00 ± 0.001	**1.77 ± 0.042**	1.00 ± 0.001	-0.02 ± 0.002	1.00 ± 0.004	**2.88 ± 0.021**
Glycerol	1.00 ± 0.006	0.04 ± 0.008	1.00 ± 0.004	**-0.43 ± 0.002**	1.00 ± 0.001	**-0.71 ± 0.003**
Threitol	1.00 ± 0.001	**0.32 ± 0.001**	1.00 ± 0.000	**-0.55 ± 0.000**	1.00 ± 0.000	**-1.20 ± 0.000**
**Others**						
Adenosine-5-monophosphate	1.00 ± 0.001	0.18 ± 0.001	1.00 ± 0.000	-0.04 ± 0.000	1.00 ± 0.000	0.14 ± 0.000
Glyceraldehyde-3-phosphate	1.00 ± 0.000	0.90 ± 0.002	1.00 ± 0.000	**-0.88 ± 0.000**	1.00 ± 0.001	**-0.94 ± 0.000**
Glucose-6-phosphate	1.00 ± 0.000	N/A	1.00 ± 0.000	N/A	1.00 ± 0.000	N/A
Fructose-6-phosphate	1.00 ± 0.000	**2.98 ± 0.000**	1.00 ± 0.000	**-2.48 ± 0.000**	1.00 ± 0.000	**1.30 ± 0.000**
Ribulose-5-phosphate	1.00 ± 0.001	-0.26 ± 0.001	1.00 ± 0.000	-0.49 ± 0.000	1.00 ± 0.000	**-1.56 ± 0.000**
Putrescine	1.00 ± 0.000	0.93 ± 0.000	1.00 ± 0.000	N/A	1.00 ± 0.000	**-2.60 ± 0.000**
Ornithine	1.00 ± 0.000	0.34 ± 0.000	1.00 ± 0.000	N/A	1.00 ± 0.001	**-2.19 ± 0.000**
Spermine	1.00 ± 0.000	N/A	1.00 ± 0.000	N/A	1.00 ± 0.000	N/A

Supplementary Table S2 B. Relative metabolite content in immature flower, open flower, immature silique and silique fully developed of WT and *ndt2-:ndt2-* plants. Metabolite amount was normalized to the internal standard ribitol before normalization to the average abundance across all stages. Values represent average ratios of normalized metabolite levels between mutant and the wild type in the corresponding tissue. Data are presented as means ± SD (n=5). Values set in bold were determined by the Student's *t* test to be significantly different ($P < 0.05$) from their corresponding WT.

	Immature flower		Open flower		Immature silique		Silique	
**Amino acids**	WT	*ndt2-:ndt2-*	WT	*ndt2-:ndt2-*	WT	*ndt2-:ndt2-*	WT	*ndt2-:ndt2-*
Alanine	1.00 ± 0.001	-0.21 ± 0.003	1.00 ± 0.002	0.05 ± 0.001	1.00 ± 0.001	0.21 ± 0.001	1.00 ± 0.001	0.51 ± 0.002
Aspartate	1.00 ± 0.049	-0.08 ± 0.062	1.00 ± 0.050	-0.03 ± 0.029	1.00 ± 0.041	**0.30 ± 0.050**	1.00 ± 0.122	0.46 ± 0.084
Asparagine	1.00 ± 0.064	-0.68 ± 0.069	1.00 ± 0.063	-0.22 ± 0.025	1.00 ± 0.042	0.30 ± 0.039	1.00 ± 0.014	0.94 ± 0.027
Arginine	1.00 ± 0.000	1.12 ± 0.000	1.00 ± 0.001	0.31 ± 0.001	1.00 ± 0.001	-0.08 ± 0.000	1.00 ± 0.000	0.34 ± 0.000
Cysteine	1.00 ± 0.033	-0.49 ± 0.026	1.00 ± 0.027	-0.16 ± 0.020	1.00 ± 0.017	0.72 ± 0.013	1.00 ± 0.016	0.59 ± 0.013
Glutamate	1.00 ± 0.133	0.02 ± 0.080	1.00 ± 0.194	-0.09 ± 0.139	1.00 ± 0.166	**0.24 ± 0.112**	1.00 ± 0.390	0.27 ± 0.069
Glutamine	1.00 ± 0.206	-0.62 ± 0.415	1.00 ± 0.374	0.51 ± 0.149	1.00 ± 0.120	1.04 ± 0.214	1.00 ± 0.078	1.04 ± 0.075
Glycine	1.00 ± 0.001	-0.34 ± 0.001	1.00 ± 0.002	-0.06 ± 0.002	1.00 ± 0.001	0.39 ± 0.002	1.00 ± 0.001	0.48 ± 0.001
Histidine	1.00 ± 0.002	-0.72 ± 0.005	1.00 ± 0.004	0.07 ± 0.002	1.00 ± 0.003	0.17 ± 0.004	1.00 ± 0.000	N/A
Homoserine	1.00 ± 0.005	-0.74 ± 0.003	1.00 ± 0.002	0.12 ± 0.001	1.00 ± 0.001	**0.91 ± 0.002**	1.00 ± 0.001	**0.99 ± 0.001**
Isoleucine	1.00 ± 0.023	-1.22 ± 0.010	1.00 ± 0.035	-0.46 ± 0.014	1.00 ± 0.014	-0.09 ± 0.011	1.00 ± 0.019	0.96 ± 0.074
Leucine	1.00 ± 0.000	-0.16 ± 0.000	1.00 ± 0.000	-0.94 ± 0.000	1.00 ± 0.000	-0.39 ± 0.000	1.00 ± 0.000	1.00 ± 0.001
Lysine	1.00 ± 0.004	-0.89 ± 0.004	1.00 ± 0.005	0.07 ± 0.004	1.00 ± 0.001	0.80 ± 0.003	1.00 ± 0.002	**0.83 ± 0.001**
Methionine	1.00 ± 0.008	-0.74 ± 0.005	1.00 ± 0.005	-0.15 ± 0.003	1.00 ± 0.002	0.75 ± 0.002	1.00 ± 0.001	0.45 ± 0.002
Proline	1.00 ± 0.346	-0.28 ± 0.336	1.00 ± 0.345	-0.46 ± 0.281	1.00 ± 0.571	-0.58 ± 0.410	1.00 ± 0.208	0.74 ± 0.296
Phenylalanine	1.00 ± 0.001	**-0.36 ± 0.003**	1.00 ± 0.003	-0.21 ± 0.002	1.00 ± 0.006	-0.33 ± 0.003	1.00 ± 0.003	0.26 ± 0.002
Serine	1.00 ± 0.034	-0.16 ± 0.034	1.00 ± 0.049	0.13 ± 0.033	1.00 ± 0.026	0.15 ± 0.028	1.00 ± 0.063	0.42 ± 0.025
Tryptophan	1.00 ± 0.001	-1.72 ± 0.001	1.00 ± 0.003	-0.29 ± 0.001	1.00 ± 0.000	0.40 ± 0.001	1.00 ± 0.000	0.97 ± 0.001
Tyrosine	1.00 ± 0.002	-0.80 ± 0.002	1.00 ± 0.004	0.15 ± 0.004	1.00 ± 0.001	0.45 ± 0.002	1.00 ± 0.004	0.51 ± 0.004
Threonine	1.00 ± 0.000	-0.28 ± 0.000	1.00 ± 0.001	0.03 ± 0.001	1.00 ± 0.000	0.12 ± 0.000	1.00 ± 0.001	0.25 ± 0.000
Valine	1.00 ± 0.020	**-0.59 ± 0.019**	1.00 ± 0.045	-0.17 ± 0.032	1.00 ± 0.012	0.03 ± 0.017	1.00 ± 0.033	0.71 ± 0.078

Suplemental table . *(Continued)*

**Organic Acids**								
Ascorbate	1.00 ± 0.001	-0.05 ± 0.001	1.00 ± 0.001	0.27 ± 0.000	1.00 ± 0.002	-0.02 ± 0.001	1.00 ± 0.001	0.22 ± 0.001
Aconitate	1.00 ± 0.000	-0.22 ± 0.000	1.00 ± 0.000	-0.01 ± 0.000	1.00 ± 0.001	-0.23 ± 0.000	1.00 ± 0.001	0.18 ± 0.001
Citrate	1.00 ± 0.032	-0.04 ± 0.022	1.00 ± 0.025	0.08 ± 0.028	1.00 ± 0.159	0.36 ± 0.036	1.00 ± 0.094	0.21 ± 0.063
Dehydroascorbate	1.00 ± 0.007	0.18 ± 0.013	1.00 ± 0.006	0.42 ± 0.006	1.00 ± 0.050	-0.36 ± 0.029	1.00 ± 0.013	-0.45 ± 0.013
Fumarate	1.00 ± 0.048	0.33 ± 0.116	1.00 ± 0.091	-0.05 ± 0.076	1.00 ± 0.122	-0.09 ± 0.063	1.00 ± 0.207	0.13 ± 0.173
Glycerate	1.00 ± 0.002	0.00 ± 0.001	1.00 ± 0.004	-0.28 ± 0.001	1.00 ± 0.004	-0.28 ± 0.003	1.00 ± 0.002	0.11 ± 0.001
Glutarate	1.00 ± 0.000	-0.06 ± 0.000	1.00 ± 0.000	0.11 ± 0.000	1.00 ± 0.000	-0.20 ± 0.000	1.00 ± 0.000	0.22 ± 0.000
GABA	1.00 ± 0.018	-0.64 ± 0.021	1.00 ± 0.033	-0.23 ± 0.018	1.00 ± 0.008	0.17 ± 0.015	1.00 ± 0.010	-0.01 ± 0.011
Lactate	1.00 ± 0.015	-0.19 ± 0.014	1.00 ± 0.011	0.01 ± 0.009	1.00 ± 0.022	0.03 ± 0.010	1.00 ± 0.010	0.65 ± 0.016
Malate	1.00 ± 0.016	-0.14 ± 0.031	1.00 ± 0.022	-0.06 ± 0.021	1.00 ± 0.051	-0.06 ± 0.020	1.00 ± 0.050	0.27 ± 0.056
Pyruvate	1.00 ± 0.000	0.01 ± 0.000	1.00 ± 0.000	-0.06 ± 0.000	1.00 ± 0.000	0.07 ± 0.000	1.00 ± 0.000	-0.13 ± 0.000
Succinate	1.00 ± 0.006	0.08 ± 0.005	1.00 ± 0.011	0.08 ± 0.010	1.00 ± 0.005	0.02 ± 0.006	1.00 ± 0.007	0.27 ± 0.002
Shikimate	1.00 ± 0.000	N/A	1.00 ± 0.000	N/A	1.00 ± 0.000	N/A	1.00 ± 0.000	N/A
**Sugars**								
Fructose	1.00 ± 0.012	-0.08 ± 0.007	1.00 ± 0.069	-0.07 ± 0.060	1.00 ± 0.183	0.31 ± 0.036	1.00 ± 0.055	0.07 ± 0.072
Fucose	1.00 ± 0.000	-0.02 ± 0.000	1.00 ± 0.001	-0.03 ± 0.001	1.00 ± 0.003	-0.25 ± 0.001	1.00 ± 0.001	-0.01 ± 0.001
Glucose	1.00 ± 0.009	0.08 ± 0.012	1.00 ± 0.029	-0.04 ± 0.021	1.00 ± 0.027	0.14 ± 0.020	1.00 ± 0.043	-0.21 ± 0.066
Galactose	1.00 ± 0.000	-0.42 ± 0.000	1.00 ± 0.001	N/A	1.00 ± 0.001	0.82 ± 0.001	1.00 ± 0.000	-0.20 ± 0.000
Glucoheptose	1.00 ± 0.060	-0.63 ± 0.061	1.00 ± 0.036	-0.26 ± 0.023	1.00 ± 0.027	-0.04 ± 0.027	1.00 ± 0.009	0.12 ± 0.007
Isomaltose	1.00 ± 0.000	-0.50 ± 0.000	1.00 ± 0.000	0.10 ± 0.000	1.00 ± 0.000	0.02 ± 0.000	1.00 ± 0.000	0.15 ± 0.000
Maltose	1.00 ± 0.000	0.10 ± 0.000	1.00 ± 0.000	0.04 ± 0.000	1.00 ± 0.000	-0.20 ± 0.000	1.00 ± 0.000	0.24 ± 0.000
Mannose	1.00 ± 0.000	-0.15 ± 0.000	1.00 ± 0.000	0.04 ± 0.000	1.00 ± 0.003	0.23 ± 0.001	1.00 ± 0.001	0.04 ± 0.001
Maltotriose	1.00 ± 0.000	0.79 ± 0.002	1.00 ± 0.000	-0.20 ± 0.000	1.00 ± 0.000	0.17 ± 0.000	1.00 ± 0.002	-0.19 ± 0.000
Raffinose	1.00 ± 0.004	-0.24 ± 0.003	1.00 ± 0.003	-0.02 ± 0.003	1.00 ± 0.010	-0.50 ± 0.007	1.00 ± 0.006	-0.14 ± 0.002
Trehalose, alpha,alpha	1.00 ± 0.000	0.00 ± 0.001	1.00 ± 0.000	0.07 ± 0.000	1.00 ± 0.001	-0.31 ± 0.001	1.00 ± 0.000	0.54 ± 0.000

Suplemental table . *(Continued)*

**Polyols**								
Erythritol	1.00 ± 0.000	-0.21 ± 0.000	1.00 ± 0.001	0.14 ± 0.001	1.00 ± 0.001	0.048 ± 0.000	1.00 ± 0.001	0.29 ± 0.001
Inositol, myo	1.00 ± 0.039	-0.08 ± 0.035	1.00 ± 0.022	0.02 ± 0.013	1.00 ± 0.057	-0.303 ± 0.032	1.00 ± 0.015	0.12 ± 0.009
Sorbitol	1.00 ± 0.000	-0.42 ± 0.000	1.00 ± 0.001	N/A	1.00 ± 0.001	0.825 ± 0.001	1.00 ± 0.000	-0.20 ± 0.000
Mannitol	1.00 ± 0.009	-0.04 ± 0.006	1.00 ± 0.083	-0.04 ± 0.079	1.00 ± 0.181	0.211 ± 0.058	1.00 ± 0.047	0.05 ± 0.062
Glycerol	1.00 ± 0.004	-0.10 ± 0.003	1.00 ± 0.002	0.06 ± 0.002	1.00 ± 0.004	-0.040 ± 0.002	1.00 ± 0.002	0.57 ± 0.003
Threitol	1.00 ± 0.000	-0.21 ± 0.000	1.00 ± 0.001	0.14 ± 0.001	1.00 ± 0.001	0.048 ± 0.000	1.00 ± 0.001	0.29 ± 0.001
**Others**								
Adenosine-5-monophosphate	1.00 ± 0.002	-0.28 ± 0.002	1.00 ± 0.001	0.30 ± 0.000	1.00 ± 0.001	0.02 ± 0.001	1.00 ± 0.002	-0.18 ± 0.001
Glyceraldehyde-3-phosphate	1.00 ± 0.002	-0.40 ± 0.002	1.00 ± 0.002	-0.11 ± 0.001	1.00 ± 0.001	-0.05 ± 0.001	1.00 ± 0.001	0.24 ± 0.001
Glucose-6-phosphate	1.00 ± 0.000	-0.10 ± 0.000	1.00 ± 0.000	0.20 ± 0.000	1.00 ± 0.000	-0.40 ± 0.000	1.00 ± 0.000	-0.22 ± 0.000
Fructose-6-phosphate	1.00 ± 0.000	-0.18 ± 0.000	1.00 ± 0.001	0.19 ± 0.001	1.00 ± 0.002	-0.30 ± 0.001	1.00 ± 0.001	0.16 ± 0.002
Ribulose-5-phosphate	1.00 ± 0.000	-0.38 ± 0.001	1.00 ± 0.001	0.11 ± 0.000	1.00 ± 0.000	0.02 ± 0.000	1.00 ± 0.000	0.10 ± 0.000
Putrescine	1.00 ± 0.007	-0.10 ± 0.009	1.00 ± 0.001	-0.19 ± 0.002	1.00 ± 0.003	0.37 ± 0.002	1.00 ± 0.000	0.35 ± 0.000
Ornithine	1.00 ± 0.004	**-1.26 ± 0.002**	1.00 ± 0.002	-0.38 ± 0.001	1.00 ± 0.002	0.76 ± 0.002	1.00 ± 0.001	**0.82 ± 0.001**
Spermine	1.00 ± 0.000	-0.54 ± 0.000	1.00 ± 0.002	0.10 ± 0.002	1.00 ± 0.001	-0.09 ± 0.001	1.00 ± 0.000	1.95 ± 0.001
