# Supplementary figures and images for: Downregulation of a Mitochondrial NAD+ Transporter (NDT2) Alters Seed Production and Germination in Arabidopsis
Source: Plant Cell Physiol. 2020 Feb 17;61(5):897–908. doi: 10.1093/pcp/pcaa017 (PMC7217668; doi:10.1093/pcp/pcaa017)

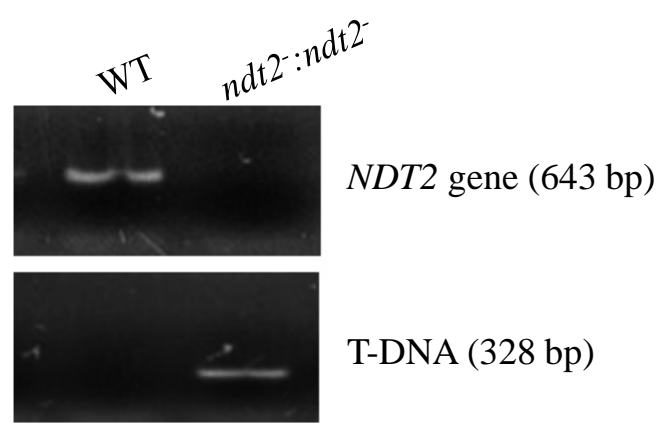

**Supplementary figure S3.** Genotyping of *ndt2<sup>-</sup>:ndt2<sup>-</sup>* mutant line.

Supplement: pcaa017_Supplementary_Data [file pcaa017_supplementary_data.zip › pcaa017-suppl_data/pcp-2019-e-00571-File009.pdf]
